# The Anatomical Remembrancer; or Complete Anatomist; Containing a Concise Description of the Bones, Ligaments, Muscles, Aud Viscera, &c. &c.

**Published:** 1845-12

**Authors:** 


*The Anatomical Remembrancer; or Complete Pocket Anatomist: containing a concise description of the Bones, Ligaments, Muscles, and Viscera, &c. &c. From the Second London edition, revised.* 18mo. pp. 245. Samuel S. & William Wood. New York, 1845.

This is a small book, and of course a very brief treatise on so extensive a subject as anatomy; nevertheless it is a good one;—one of the best manuals on the subject in the English language.

Every part and tissue are mentioned—bones, muscles, ligaments, viscera, nerves, blood vessels and absorbents, and their proper distribution, arrangement of the fasciæ, organs of sense and organs of generation. It is not intended or calculated to give a full and comprehensive account of anatomy, such as a student should have in mastering the subject, but it is well adapted, by the clearness and brevity of the account given under each head, to subserve the purposes of a *remembrancer,*—a work to be picked up in a hasty moment in order to recall to the mind the material points on which a student may desire to be refreshed, without the trouble of reading over a more elaborate treatise.

